# Sono-Gas-Mediated Precise Stiffness Remodeling for Triple-Negative Breast Cancer Mechanical Immunotherapy

**DOI:** 10.34133/bmr.0207

**Published:** 2025-05-15

**Authors:** Yaqin Hu, Long Cheng, Xun Guo, Min Zheng, Wei Zhang, Xingyue Wang, Rui Tang, Qiaoqi Chen, Yuan Guo, Yang Cao, Zhigang Wang, Haitao Ran

**Affiliations:** ^1^ Department of Ultrasound, the Second Affiliated Hospital of Chongqing Medical University, Chongqing 400010, People’s Republic of China.; ^2^ Chongqing Key Laboratory of Ultrasound Molecular Imaging and Therapy, the Second Affiliated Hospital of Chongqing Medical University, Chongqing 400010, People’s Republic of China.; ^3^ Department of Ultrasound, Affiliated Hospital of Hubei University of Arts and Science, Xiangyang, Hubei Province 441021, People’s Republic of China.

## Abstract

Triple-negative breast cancer (TNBC) is a highly invasive cancer, and its poor therapeutic outcomes are often associated with the mechanical properties of the tumor microenvironment, which is characterized by altered extracellular matrix (ECM) flexibility and increased stiffness. Herein, a mechanical immunomodulator, namely, red blood cell membrane-IR780-L-arginine nanoparticles (R-I-LA NPs), was designed to precisely regulate the stiffness of the ECM for mechanical immunotherapy of TNBC. In tumor cells, the low-intensity focused ultrasound activates R-I-LA NPs to produce reactive nitrogen species, which damages tumor cells and remodels the stiffness of ECM. Meanwhile, the softened ECM can normalize the tumor vasculature to alleviate hypoxia and increase the production of reactive oxygen species, thereby enhancing the efficacy of sonodynamic therapy and stimulating immunogenic cell death. Additionally, R-I-LA NPs stimulate the immune system and suppress pulmonary metastasis. Overall, this study offers a distinctive “sono-gas-mediated mechanical immunity” strategy for ECM regulation, potentially overcoming current TNBC therapy limitations.

## Introduction

Triple-negative breast cancer (TNBC) is a subtype of breast cancer with the worst prognosis due to lack of therapeutic targets and the emergence of resistance to chemotherapy and immunotherapy [1,2]. Recently, nanomedicine-based immunotherapies, such as photothermal therapy and ferroptosis, have shown great promise in the treatment of TNBC [3]. However, the available strategies overlook the biological stress factors in the tumor microenvironment (TME) [4]. The unique physiological characteristics of TNBC create the tumor mechanical microenvironment (TMM), where increased extracellular matrix (ECM) stiffness not only influences the death of cancer cells but also disrupts the integrity of the cancer-immunity cycle, such as interfering with the release of antigens from cancer cells, the antigen presentation by dendritic cells (DCs), and the migration of T cells [5–8]. Currently, strategies targeting the ECM have been proven to effectively improve the therapeutic outcomes of TNBC [9,10]. However, given that the ECM is not specific to tumor cells, nonselective systemic administration will inevitably lead to adverse reactions such as activation of nontarget organs [11,12]. Therefore, an integrated therapeutic approach that precisely regulates ECM to dismantle the mechanical barriers is urgently needed to enhance the immune response rate in TNBC.

Reactive nitrogen species (RNS), which are a group of highly oxidizing free radicals and nitro-compounds, are produced through the interaction of nitric oxide (NO) and reactive oxygen species (ROS) [13,14]. RNS exhibit important oxidative activity, which is capable of directly oxidizing and damaging cells, leading to immunogenic cell death (ICD) [15]. Additionally, RNS activate matrix metalloproteinases (MMPs) that facilitate the degradation process of type I collagen, which is a major component of ECM**,** thereby playing a pivotal role in improving tumor mechanical forces [16]. These findings highlight the need for a platform that assembles stimuli-responsive ROS generators and NO donors to ensure the synchronous generation of these 2 substances in space and time, which remains a challenge for the effective formation of RNS within the tumor. Therefore, this therapeutic strategy of precise temporal and spatial control of RNS generation to regulate ECM mechanical properties remains largely unexplored.

Numerous studies have demonstrated that the controlled generation of ROS in situ can be achieved by targeting drug delivery system and adjusting the power and time of ultrasound irradiation [17,18]. Thus, sonodynamic therapy (SDT) offers a safe, noninvasive, and spatiotemporally controllable approach to modulate ECM [19,20]. L-arginine (LA) is an essential amino acid for the human body, possessing good biosafety, and is a reliable donor**.** SDT-induced ROS, in conjunction with inducible nitric oxide synthase (iNOS) within cells, catalyze the conversion of LA to NO. The NO then reacts with ROS to generate RNS in situ within tumors [21]. This sono-gas-mediated approach allows for controlled production of RNS in the tumor region, leading to ECM softening and subsequently increasing oxygen delivery to the tumor tissue. The increase in oxygen content enhances the effectiveness of SDT, achieving a self-enhanced feedback pathway. Self-enhanced SDT-induced ROS and RNS storms expose tumor cells to large amounts of antigen and damage-associated molecular patterns (DAMPs), causing tumor ICD [22,23]. Thus, sono-activated immunomodulators are expected to improve the efficacy of TNBC immunotherapy by breaking tumor mechanical barrier.

In this study, we developed R-I-LA NPs, a mechanical immunomodulator, combining an IR780 (a sonosensitizer) and LA (a NO donor) within red blood cell membrane-coated biomimetic nanoparticles. This innovative sono-gas-mediated mechanical immunotherapy improves TMM by precisely regulating ECM stiffness using low-intensity focused ultrasound (LIFU). The red blood cell membrane’s evasion characteristics facilitate the efficient delivery of red blood cell membrane-IR780-L-arginine nanoparticles (R-I-LA NPs) to tumor sites [24]. IR780 possesses the characteristics of photoacoustic and fluorescence dual-modality imaging, which can be used to guide therapy, enhancing the precise generation of SDT. Furthermore, SDT-induced ROS catalyze the conversion of LA to NO, thereby cascading the production of RNS. The RNS-mediated ECM softening can increase the oxygen influx, cresting a ROS storm that effectively contributes to ICD and the release of tumor-associated antigens. The influx of immune cells into the TME enhances antigen presentation by DCs and increases T cell cytotoxicity, thereby augmenting the antitumor response and further stimulating the immune response. By precisely regulating the ECM mechanics through sono-gas-mediated immunotherapy, we can create a more favorable TME, leading to synergistic improvements in TNBC immunotherapy.

## Materials and Methods

### Materials

All reagents employed in this study were of analytical grade. Polylactic glycolic acid (PLGA; lactide:glycolide = 50:50, molecular weight 12,000 Da) was purchased from Shandong Taigang Bioengineering Co., China. IR780, a fluorescent small molecule derived from seven-methylchrysin, was bought from China Bailingwei Co. LA, the Griess reagent kit, mitochondrial fluorescence probe, JC-1 assay kit, and fluorescent dyes including 4′,6-diamidino-2-phenylindole (DAPI) and 1,1′-dioctadecyl-3,3,1′,3′-tetramethylindocarbocyanine chloride (DiI) were purchased from China Bilingtian Co. Polyvinyl alcohol (PVA; molecular weight, 25,000 Da) was bought from Sigma-Aldrich (Shanghai). The single-line oxygen fluorescence probe (SOSG) was purchased from Thermo Fisher Scientific in the United States. The Cell Counting Kit-8 (CCK-8) was acquired from MedChemExpress (Monmouth Junction, NJ). The live/dead cell staining kit containing calcein AM and propidium iodide (PI) was purchased from Tokyo East Nippon Co., Ltd. Specific culture media for 4T1 cells and RAW264.7 macrophages were purchased from Wuhan PuroScience Co., Wuhan, China.

### Synthesis of I-LA NPs

The I-LA nanoparticles were synthesized using a conventional double-emulsification technique. Briefly, 50 mg of PLGA, 2 mg of IR780, and 20 mg of LA were accurately weighed. Initially, PLGA and IR780 were dissolved in 2 ml of dichloromethane; LA solution was using 200 μl of deionized water and mixed with dichloromethane. In a nitrogen-rich environment, the resulting solution was sonicated with a sonicator (40 W for 2 min), and then added to 8 ml of PVA and further sonicated for 2 min. Finally, 15 ml of a 2% isopropanol solution was added and magnetically stirred for 5 h to remove dichloromethane. The resultant suspension was repeatedly centrifuged at 10,000 rpm for 8 min to isolate the I-LA particles, which were then stored at 4 °C for subsequent experiments. Utilizing the same protocol, alternative formulations such as LA NPs (PLGA encapsulated with LA) and I NPs (PLGA encapsulated with IR780) were prepared by substituting specific components accordingly. Moreover, DiI-labeled PLGA nanospheres were fabricated using the same protocol as above. However, DiI was integrated into the CHCl_2_ mixture.

### Extraction of red cell membrane and synthesis of R-I-LA NPs

Fresh mouse blood samples were collected into an EDTA-anticoagulant tube and centrifuged at 4 °C (3,000 rpm for 5 min) using a low-temperature centrifuge to obtain red blood cells from the precipitate. The cells were washed 3 times with 1× phosphate-buffered saline (PBS), and suspended in 0.2× PBS to induce hypotonic lysis. Subsequently, the cells were centrifuged at 11,000 rpm for 5 min thrice to remove membrane debris, yielding a pale pink red blood cell membrane. The red blood cell membrane was sonicated using a sonicator to disrupt the cells (40 W for 2 min). Next, 1 mg of I-LA nanoparticles was added to 1 ml of whole blood repeatedly extruded using a lipid extruder. The resulting mixture was centrifuged and washed 3 times to eliminate excess red blood cell membranes to obtain R-I-LA NPs.

### Characterization of R-I-LA NPs

The I-LA NPs and R-I-LA NPs were dried on a copper mesh and examined under a transmission electron microscope (Hitachi H-7600, Tokyo, Japan) to examine their morphology and size. The size, zeta potential, and dispersibility of the I-LA NPs and R-I-LA NPs were analyzed using a Malvern particle size analyzer (Nano ZS90, Malvern Instrument, UK). Variations in size and dispersibility of R-I-LA NPs suspended in PBS were monitored for 1 week. The proteins expressed on the surface of red blood cells coated with the nanoparticles were detected by Western blot analysis. The R-I-LA NPs were dissolved in methanol and then subjected to UV absorption spectrum measurement. The drug loading capacity of IR780 was determined from a calibration curve. Various concentrations of LA solution were prepared and analyzed by liquid chromatography–mass spectrometry, and their calibration curves were established. Finally, the drug loading capacity of LA was calculated accordingly.

### Detecting ROS and NO production capacity in vitro

A series of concentrations of R-I NPs and R-I-LA NPs solutions (equivalent to IR780 concentration of 4, 12, and 20 μg/ml) were mixed with diluted SOSG solution (10 μg/ml in methanol) evenly, and irradiated with LIFU for different times (0, 30, 60, 90, 120, 150, and 180 s). The fluorescence intensity of the solutions was detected using a multimode reader (SpectraMax M, Molecular Devices, USA). A NO concentration calibration curve was prepared following the instructions on the Griess reagent kit. Different concentrations of 0.5 mg/ml R-I-LA NPs, R-I NPs, and R-LA NPs were prepared in 3 ml of a spectrophotometer cuvette using deionized water, into which 100 μl of Griess I and Griess II reagents were added. The samples were irradiated with LIFU for different times (0, 30, 60, 120, and 180 s) and the fluorescence intensity was recorded using a UV–visible spectrophotometer.

### Photoacoustic and fluorescence imaging performance in vitro

The full-spectrum scan of the photoacoustic imaging system (Vevo LAZR, Canada) was employed to identify the optimal wavelength range for the R-I-LA NPs solution at a concentration of 50 μg/ml. Next, a series of concentrations of R-I-LA NPs solution (6.25, 12.5, 25, 50, and 75 μg/ml) were added into the gel module and irradiated with the identified optimal excitation wavelength. Photoacoustic images were acquired and the corresponding photoacoustic signal values for different concentrations of R-I-LA NPs (0.5, 1.0, 1.5, 2.0, and 2.5 mg/ml) were analyzed. Finally, R-I-LA NPs were deposited into wells on a plate and imaged using a fluorescence imaging system (Fx7 Ir Spectra; Vilber Lourmat, France), with fluorescence signals analyzed via Indigo software.

### The cellular uptake and mitochondria-targeting capability of R-I-LA NPs

4T1 cells were cultured with the 4T1 cell-specific culture medium at 37 °C in a 5% CO_2_ incubator. Initially, the cells were seeded into laser confocal dishes and 12-well plates, with 50,000 cells/well. The cells were allowed to adhere for 24 h, and the culture medium was discarded. They were washed once with PBS mixed with either R-I-LA or I-LA nanoparticles (0.1 mg/ml) at specific time points. This was followed by trypsin digestion and centrifugation, and finally resuspended for further analysis. The proportion of nanoparticle-ingesting cells was quantified using flow cytometry (FCM). For confocal laser scanning microscopy (CLSM) observations, the cells in the dishes were fixed with paraformaldehyde (4%) for 15 min and stained with DAPI (200 μl) for another 15 min to visualize nuclei. The nanoparticle uptake under CLSM conditions was assessed. To further evaluate mitochondrial targeting capabilities, identical procedures were followed in which mitochondria-specific dye was added into the confocal dish for 20 min. The cells were washed to remove excess dye and examined using the CLSM to observe the colocalization of nanoparticles and mitochondria.

### R-I-LA NPs evade macrophage phagocytosis

RAW264.7 macrophages were cultured in a macrophage-specific medium and incubated at 37 °C in a 5% CO_2_ atmosphere. The cells were seeded at a density of 50,000 cells per well in laser confocal dishes. They were allowed to adhere for 24 h and then mixed with 1 ml of R-I-LA nanoparticles and I-LA nanoparticles (at a concentration of 0.1 mg/ml). They were further incubated for 4 h, fixed with formaldehyde for 15 min, and stained with 200 μl of DAPI dye for 15 min to visualize the cell nuclei. The samples were then analyzed using CLSM to evaluate the nanoparticle uptake by macrophages.

### Detection of cellular production of ROS, NO, and RNS

The cells were divided into 8 groups: control group (normoxic/hypoxia), R-I NPs + LIFU (normoxia/hypoxia), R-LA NPs + LIFU (normoxia/hypoxia), and R-I-LA NPs + LIFU (normoxia/hypoxia). A total of 50,000 4T1 cells were cultured in laser confocal dishes and 12-well plates at a density of 50,000 cells per well. Cells designated for hypoxic conditions were transferred to hypoxic sealed boxes after an initial adhesion period of 8 h. This was followed by incubation for 24 h, each well in the control group was added 1 ml of the requisite nanoparticles at a concentration of 0.1 mg/ml, while control wells were supplemented with 1 ml of basic culture medium RPMI-1640. After 4 h, excess nanoparticles were removed by washing with PBS, and each well was treated with either DCFH-DA for ROS detection or DAF-FM DA for NO detection or an appropriate reagent for RNS detection and incubated for 20 min. LIFU irradiation treatment was performed at a power setting of 2 W/cm^2^ in pulse mode for 3 min. The cells were imaged using a laser confocal microscope in darkness in their respective confocal dishes. Meanwhile, those from the wells were digested with trypsin, transferred to 1.5-ml EP tubes and centrifuged at 1,000 rpm. The resulting cell suspension was diluted and analyzed via FCM.

### Detection of mitochondrial membrane potential

The experimental groups and treatment conditions for examining the mitochondrial membrane potential using JC-1 staining were performed using the same protocol as previous assays. Briefly, 4 h after treatment, excess nanoparticles were washed off with PBS, and cells were incubated with 1 ml of JC-1 staining solution (10 μM) for 20 min. Cells in confocal dishes were imaged under a laser confocal microscope in a dark environment. Meanwhile, cells from well plates were digested with trypsin, transferred to 1.5-ml EP tubes, and centrifuged at 1,000 rpm to pellet the cells. The samples were then diluted and analyzed using FCM.

### Antitumor efficacy in vitro

Initially, the cytotoxic effects of R-I-LA NPs on 4T1 cells were determined using the CCK-8 assay. Briefly, 10,000 cells per well were seeded in a 96-well plate and randomly divided into 6 experimental groups: control group (normoxia/hypoxia), LIFU-only (normoxia/hypoxia), R-I-LA NPs (normoxia/hypoxia), R-LA NPs + LIFU (normoxia/hypoxia), R-I NPs + LIFU (normoxia/hypoxia), and R-I-LA NPs + LIFU (normoxia/hypoxia). The cells were incubated for 24 h and then added with 200 μl of nanoparticles at a concentration of 0.1 mg/ml. They were incubated for 4 h before treatment with LIFU at a power density of 2 W/cm^2^ in pulsed mode for 3 min. Posttreatment, the cells were incubated under controlled conditions for 2 h, and each well was supplemented with 200 μl of a 10% CCK-8 reagent. The absorbance was measured at a wavelength of 450 nm using a multifunctional enzyme-linked immunosorbent assay (ELISA) reader after the formation of an orange coloration. The cell viability and apoptosis were examined using the live/dead double staining via CLSM and the cell apoptosis was determined using the FCM. The cytotoxicity of blank PLGA nanoparticles was evaluated by CCK-8. The cells were incubated with blank PLGA nanoparticles **(**0.1 mg/ml**)** for 72 h to detect cell activity.

### Establishment of animal model

Female BALB/c mice (6 weeks old, approximately 16 g) were purchased from Hunan Ensville Co., Ltd. and housed at the Animal Experiment Center of Chongqing Medical University. The 4T1 mouse mammary carcinoma cells were digested with trypsin and centrifuged at 2,000 rpm for 5 min. The supernatant was discarded and the cell pellet was resuspended in sterile PBS to a final concentration of 1 million cells per μl. Each mouse was injected with 100 μl of this cell suspension on the right fourth mammary gland. The mice were housed in the IVC-level animal experiment facilities while being monitored regularly to assess tumor growth. When tumor volume reached a volume of about ~100 mm^3^, further treatment protocols were initiated. Tumor volume was calculated using the formula *V* = 0.52 × *L* × *W*^2^, where *L* is the tumor’s length and *W* is its width. Tumor growth was monitored at intervals with calipers. When the volume surpassed 1,500 mm^3^ or the tumor burden jeopardized animal welfare, euthanasia was executed via CO₂ inhalation. This study adhered to standardized tumor volume protocols to guarantee reproducibility and ethical compliance. The maximal tumor size in this study was not exceeded. All experimental protocols were approved by the Animal Ethics Committee of the Second Affiliated Hospital of Chongqing Medical University (Review No. 2021-265).

### Photoacoustic and fluorescence imaging performance in vivo

Mice in the TNBC model group were randomly assigned to 2 groups (*n* = 3) and anesthetized with pentobarbital. They were then preinjected for imaging. Subsequently, R-I-LA NPs and I-LA NPs (200 μl, 2.5 mg/ml) were administered via the tail vein at various time points (2, 4, 8, 24, and 48 h). At the 48-h mark, the mice were anesthetized and euthanized to collect tumors and other vital organs (heart, liver, spleen, lung, and kidney) for ex vivo fluorescence imaging. The acquired images were processed and analyzed utilizing Indigo software. A similar methodology was employed to assess the photoacoustic imaging effects of nanoparticles in vivo at different time intervals.

### ECM degradation and the rectification of the hypoxic microenvironment in tumors

After successful establishment of the TNBC mouse tumor model, the mice were randomly allocated into 6 groups (*n* = 5): control, R-I-LA NPs, R-I NPs + LIFU, R-LA NPs + LIFU, R-I-LA NPs + LIFU, and R-I-LA NPs + LIFU + uric acid. Mice in the R-I-LA NPs received ultrasound combined with uric acid group administered via drinking 1 day before treatment followed by gastric lavage administration of uric acid 4 h before treatment. All other groups received identical treatments. Following 24 h of treatment, tumors were excised and embedded in paraffin; 10-μm-thick sections were prepared and then incubated at room temperature for 1 h with mouse anti-3-nitrotyrosine (3-NT) antibody, while cell nuclei were stained purple with hematoxylin. Concurrently, immunofluorescence staining was conducted for hypoxia markers HIF-1α and hypoxia as well as type I collagen for ECM examination. Furthermore, Western blot analysis was performed to evaluate the expression levels of MMPs in the ECM of tumor cells, utilizing rabbit anti-MMP-1 and MMP-2 specific antibodies (Proteintech, China). The enzymatic activity of MMPs was determined through in situ enzymatography using the EnzChek Collagenase/Elastase Assay Kit (Thermo Fisher Scientific, Shanghai, China).

### Antitumor efficacy in vivo

The successfully established TNBC in situ tumor model mice were randomly assigned to 5 groups (*n* = 5): control, R-I-LA NPs, R-I NPs + LIFU, R-LA NPs + LIFU, and R-I-LA NPs + LIFU. Each group was injected with nanoparticles at a dose of 200 μl at a concentration of 2.5 mg/ml. Mice in the control group received an equivalent volume of normal saline. The LIFU was applied at a power setting of 3 W/cm^2^ using a pulse pattern with intervals of 2 s for 8 min. The treatment schedule was organized as follows: nanoparticle injections were administered on the day successful modeling was confirmed, followed by LIFU irradiation on the next day, and on days 5 and 9. Body weight and tumor size were measured daily throughout the treatment period. At the end of the observation period on day 18, mice were anesthetized and euthanized, after which tumors were photographed and weighed. Histological analysis, using hematoxylin and eosin (H&E) staining, was performed to assess morphological changes in key organs (heart, liver, spleen, lungs, and kidneys) and tumors across all groups. Tumor tissues were also subjected to terminal deoxynucleotidyltransferase dUTP nick end labeling (TUNEL) assay and proliferating cell nuclear antigen (PCNA) immunofluorescence staining.

### Biosafety of R-I-LA NPs in vivo

Thirty BALB/c mice were randomly assigned to 6 groups (*n* = 5), and 200 μl of R-I-LA NPs suspended in physiological saline was administered subcutaneously on days 1, 3, 7, 14, and 28. Blood samples were collected from the mice at day 28 postinjection for biochemical profiling and complete blood counts. Major organs (heart, liver, spleen, lung, and kidney) were harvested and subjected to H&E staining for pathological analysis.

Thirty tumor-bearing BALB/c mice were randomly assigned to 6 groups (*n* = 3), and PLGA nanoparticles (200 μl, 2.5 mg/ml) suspended in physiological saline were administered subcutaneously on days 1, 3, 7, 14, and 21. On the 21st day after injection, the tumor tissues of the mice were collected for ELISA detection of TNF-α and IL-6 levels.

### Statistical analysis

All quantitative data were presented as mean ± standard deviation (SD). Statistical analyses were conducted using GraphPad Prism version 10.2.1. Differences between 2 groups were compared using Student’s 2-tailed *t* test, while one-way analysis of variance (ANOVA) was employed to evaluate differences among multiple groups (*P < 0.05, **P < 0.01, ***P < 0.001, ****P < 0.0001).

## Results and Discussion

### Synthesis and characterization of R-I-LA NPs

**Fig. 1.**
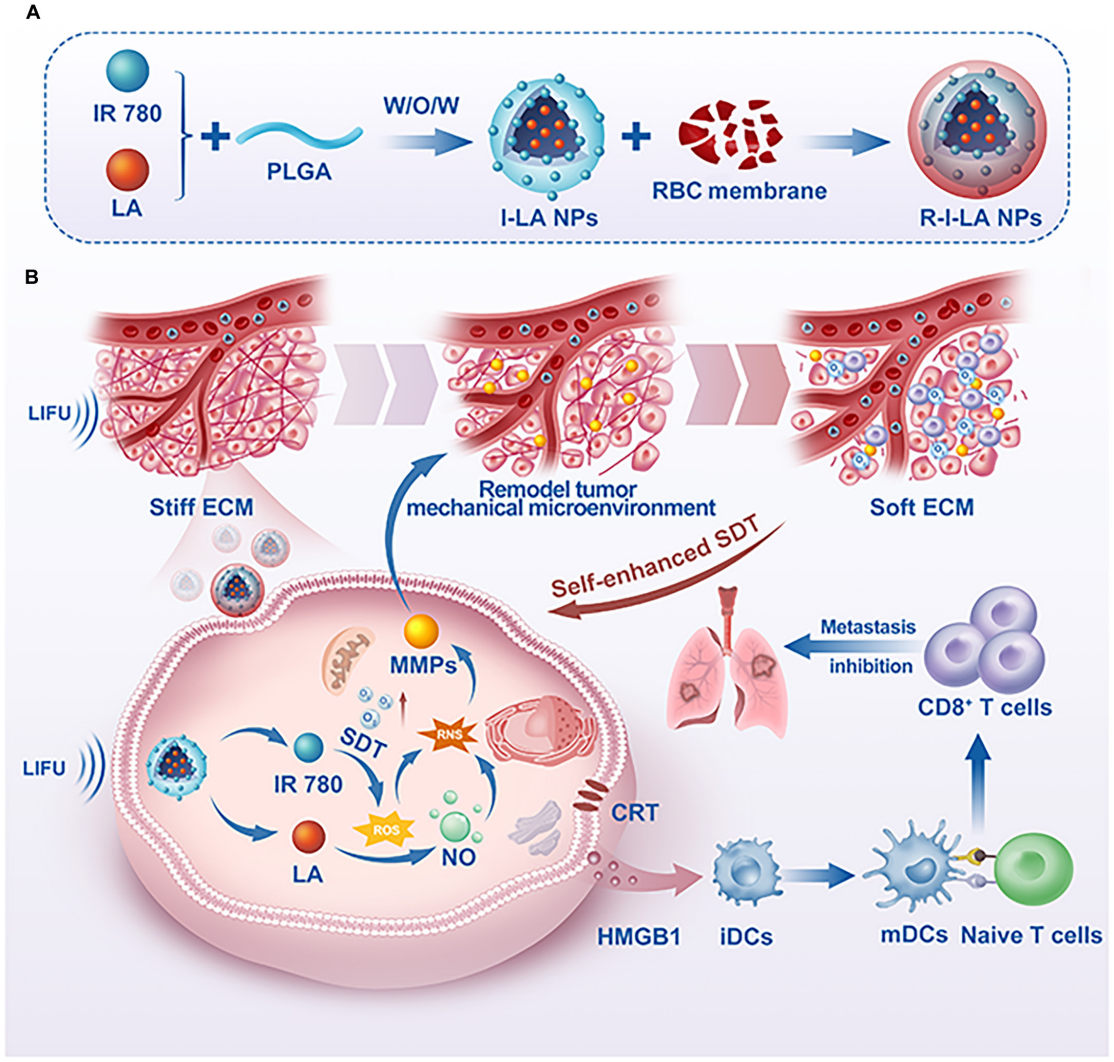
(A) The preparation process of R-I-LA NPs. (B) The sono-activated biomimetic nanoparticles (R-I-LA NPs) generate reactive nitrogen species (RNS) through a cascade reaction activated using low-intensity focused ultrasound (LIFU). RNS effectively remodels the tumor mechanical microenvironment (TMM) by reducing extracellular type I collagen, which improves the effectiveness of sonodynamic therapy (SDT) and strengthens the immune response against TNBC.

**Fig. 2.**
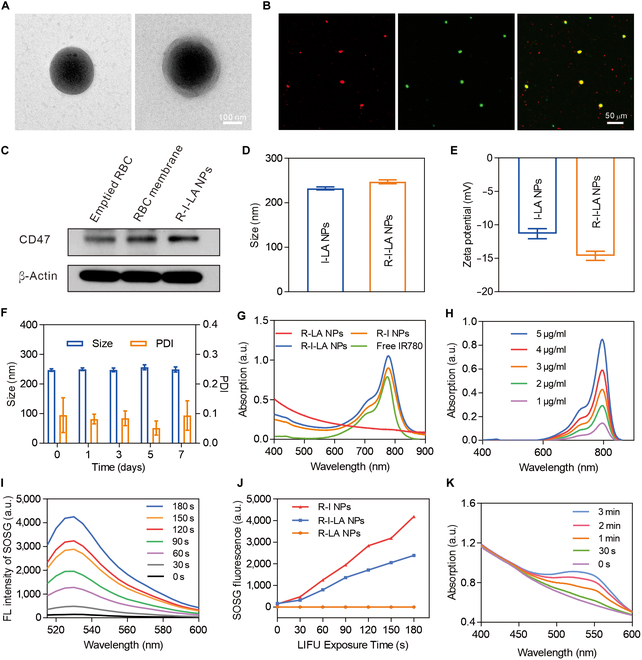
Characterization of R-I-LA NPs. (A) TEM images of I-LA NPs and R-I-LA NPs. (B) CLSM images of R-I-LA NPs. (C) Western blotting analysis of CD47. (D) Size distribution of I-LA NPs and R-I-LA NPs. (E) Zeta potential of I-LA NPs and R-I-LA NPs. (F) Stability of R-I-LA NPs based on DLS and PDI. (G) UV–vis–NIR absorbance spectra of different NPs (free IR780, R-LA NPs, R-I NPs, and R-I-LA NPs). (H) UV–vis–NIR absorbance spectra of free IR780 at elevated concentrations. (I) SOSG fluorescence intensity of R-I-LA NPs during LIFU irradiation. (J) LIFU irradiation time-dependent singlet oxygen (^1^O_2_) yield of different NPs (R-LA NPs, R-I NPs, and R-I-LA NPs). (K) UV–vis absorption of the R-I-LA NPs solution after adding Griess reagent during LIFU irradiation. The experiments were repeated thrice independently. ANOVA with Tukey’s post-hoc test.

We successfully synthesized R-I-LA NPs using dual emulsification and lipid extrusion techniques (Fig. 1A). IR780, a lipophilic small-molecule dye, is encapsulated within a PLGA shell, with the LA serving as the hydrophilic core of the PLGA nanoparticles. The fluidity of the cell membrane facilitates self-assembly around the nanoparticle sphere after an ultrasound fragmentation; additionally, mechanical forces exerted by ultrasound promote the wrapping of nanoparticles into phospholipid layers. Transmission electron microscopy (TEM) images reveal that I-LA NPs were displayed as black spherical structures, whereas R-I-LA NPs additionally displayed faint membrane-like structures on top of these spheres, which indicated the protective property of the red blood cell membrane (Fig. 2A and B) [25]. To visualize fusion between red blood cell membranes and I-LA NPs, we labeled I-LA NPs with DiI (red fluorescence) and red blood cell membranes with DiO (green fluorescence) through ultrasound emulsification; subsequent CLSM revealed complete overlap between the components resulting in a yellow fluorescence (Fig. 2B). Furthermore, to verify successful coating with red blood cell membranes on the nanoparticles, we examined the presence of CD47 on the surface of R-I-LA NPs using Western blotting (Fig. 2C) [26]. These findings validate both the successful synthesis of R-I-LA NPs and their capacity to evade macrophage phagocytosis [27].

We evaluated the basic physicochemical properties of R-I-LA NPs. The results indicate that the size of R-I-LA NPs (247.27 ± 4.38 nm) was slightly larger than that of I-LA NPs (232.3 ± 3.31 nm) due to the encapsulation of the red blood cell membranes (Fig. 1D). The Zeta potentials of I-LA NPs and R-I-LA NPs were −11.33 ± 0.75 mV and −14.63 ± 0.68 mV, respectively, with the potential of nanoparticles negatively increased when the red blood cell membrane was coated (Fig. 2E). The particle size and surface charge facilitate the penetration of the vascular space, as well as enabling the particles to evade the phagocytotic activity of the reticuloendothelial system, thus extending the blood circulation time of the nanoparticles. Subsequent examinations revealed that the particle size of R-I-LA NPs and the aggregation index (PDI) did not change significantly within a week, indicating that the particles possessed good dispersion and stability under physiological conditions (Fig. 2F). The absorbance of IR780 at different concentrations was measured using ultraviolet–visible spectroscopy (UV–vis), with the results revealing a linear relationship between the absorbance of IR780 at 780 nm and the concentration (Fig. 2H). Consequently, the encapsulation efficiency of IR780 in R-I-LA NPs was determined to be 77% ± 3.86% and the drug loading capacity was 2.86% ± 0.08% based on the standard curve of IR780 (Figs. S1 and S2). Additionally, the standard curve of LA was drawn using a high-performance liquid chromatography with the findings revealing that the encapsulation efficiency of LA in R-I-LA NPs was 41.4% ± 3.56%, while the drug loading capacity was 15.4% ± 0.69% (Figs. S2 and S3). UV–vis–NIR spectrophotometry revealed that IR780, R-I NPs, and R-I-LA NPs had a characteristic absorption peak of IR780 at 780 nm, compared with a smooth curve for R-LA NPs lacking IR780. This indicated that IR780 was successfully loaded on the nanoparticles (Fig. 2G) [28,29].

### Detection of extracellular ROS and NO induced by R-I-LA NPs

To assess the ability of R-I-LA NPs to induce ROS production in vitro, SOSG, which is a ROS detection reagent, was initially added to the R-I-LA NPs solution—the reagent and the solution were at the same concentration—and then irradiated it with LIFU. The results showed that the fluorescence intensity of SOSG at 525 nm was markedly enhanced with an increase of irradiation time (Fig. 2I and Fig. S4). These results indicate that LIFU initiated and controlled ROS production [30]. Next, we investigated the ROS production capacity of different nanoparticle solutions of R-I NPs, R-LA NPs, and R-I-LA NPs after exposure to LIFU irradiation. The results showed that with an increase of irradiation time, there was no ROS production in the R-LA NPs group due to the absence of the sonosensitizer IR780, whereas ROS production in the R-I NPs and R-I-LA NPs groups was positively correlated with the irradiation time of LIFU (Fig. 2J). However, the ROS production in the R-I-LA NPs group was lower compared to that in the R-I NPs group, due to consumption of some ROS in R-I-LA NPs in the process of oxidizing LA to produce NO. Subsequently, we investigated the capability of R-I-LA NPs to produce NO using an NO detection kit. The results indicated that with the extension of LIFU irradiation time, the production of NO increased (Fig. 1K and Figs. S5 and S6) [31]. Moreover, the NO amount produced by R-I-LA NPs under normoxic conditions was much higher than that under hypoxic conditions (Fig. S6). This is because the production of NO by LA is primarily attributed to the pro-oxidant effects of the ROS during LIFU excitation; NO production is not only dependent on LA, but also influenced by the availability of oxygen, which plays a vital role in the production of ROS.

### Intracellular uptake of R-I-LA NPs

To investigate the targeting performance of R-I-LA NPs on 4T1 cells, we observed the colocalization of DiI-stained LA NPs, I-LA NPs, and R-I-LA NPs with DAPI-stained 4T1 nuclei under CLSM.

**Fig. 3.**
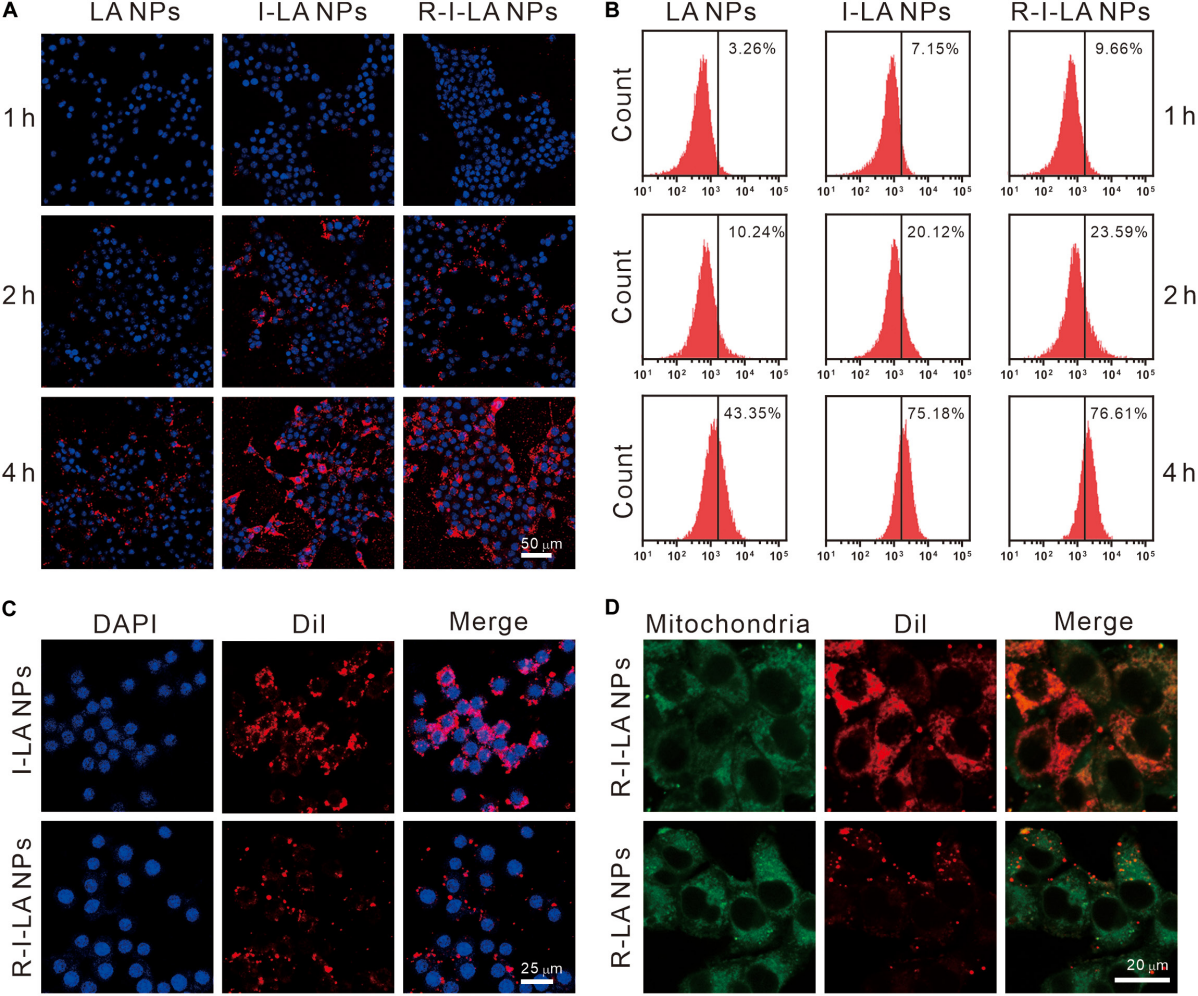
Evaluation of intracellular uptake. (A) CLSM images of DiI-labeled different NPs (LA NPs, I-LA NPs, and R-I-LA NPs) colocalized with 4T1 cells. (B) FCM analysis of 4T1 cells after coincubation with different NPs for various times. (C) CLSM images of Raw264.7 mouse macrophage cells treated with I-LA NPs or R-I-LA NPs. (D) CLSM images of DiI-labeled R-LA NPs or R-I-LA NPs colocalized with Mito-Tracker green in 4T1 cells. The experiments were repeated thrice independently. ANOVA with Tukey’s post-hoc test.

The results indicated that with an increasing co-incubation time (1, 2, and 4 h), the phagocytotic uptake of I-LA NPs and R-I-LA NPs by 4T1 was significantly greater than that of LA NPs, indicating that IR780 enhanced the phagocytotic efficiency of tumor cells toward the nanoparticles. However, there was no significant difference in phagocytotic uptake of the I-LA NPs and R-I-LA NPs groups (Fig. 3A). FCM was used to quantitatively analyze the phagocytotic uptake ratio of each group of nanoparticles by 4T1 cells. The results showed that after 4 h of incubation, the cellular uptake ratio of I-LA NPs and R-I-LA NPs was 72.78% ± 5.35% and 72.27% ± 3.38%, respectively (Fig. 3B and Fig. S7), whereas there was no statistical difference between I-LA NPs and R-I-LA NPs groups. While the coating of erythrocyte membrane reduced the uptake of nanoparticles by tumor cells, when we incubated RAW264.7 mouse macrophages with I-LA NPs and R-I-LA NPs for 4 h, we revealed that the uptake of R-I-LA NPs by macrophages was significantly lower compared to that of I-LA NPs. These results indicate that the encapsulation of erythrocyte membrane provides the nanoparticles with the ability to evade macrophage phagocytotic activity, which is primarily attributed to the erythrocyte membranes that highly express CD47 protein, which, upon binding to the signal regulatory protein alpha (SIRPα) receptor on the surface of RAW264.7 macrophages, triggers a “self-recognition” signal that inhibits the phagocytic activity of macrophages (Fig. 3C). Meanwhile, the results of mitochondrial targeting experiments showed that R-I-LA NPs loaded with IR780 exhibited excellent colocalization performance with cellular mitochondria (Fig. 3D). This effect is attributed to IR780 being a lipophilic cationic compound, a property that enables R-I-LA NPs to easily penetrate the cell membrane and mitochondrial bilayer. This enhanced permeability is driven by the elevated membrane potential of tumor cells and the involvement of organic anion transporter peptides, which aid in the uptake of the nanoparticles.

### Therapeutic efficacy of R-I-LA NPs in vitro

**Fig. 4.**
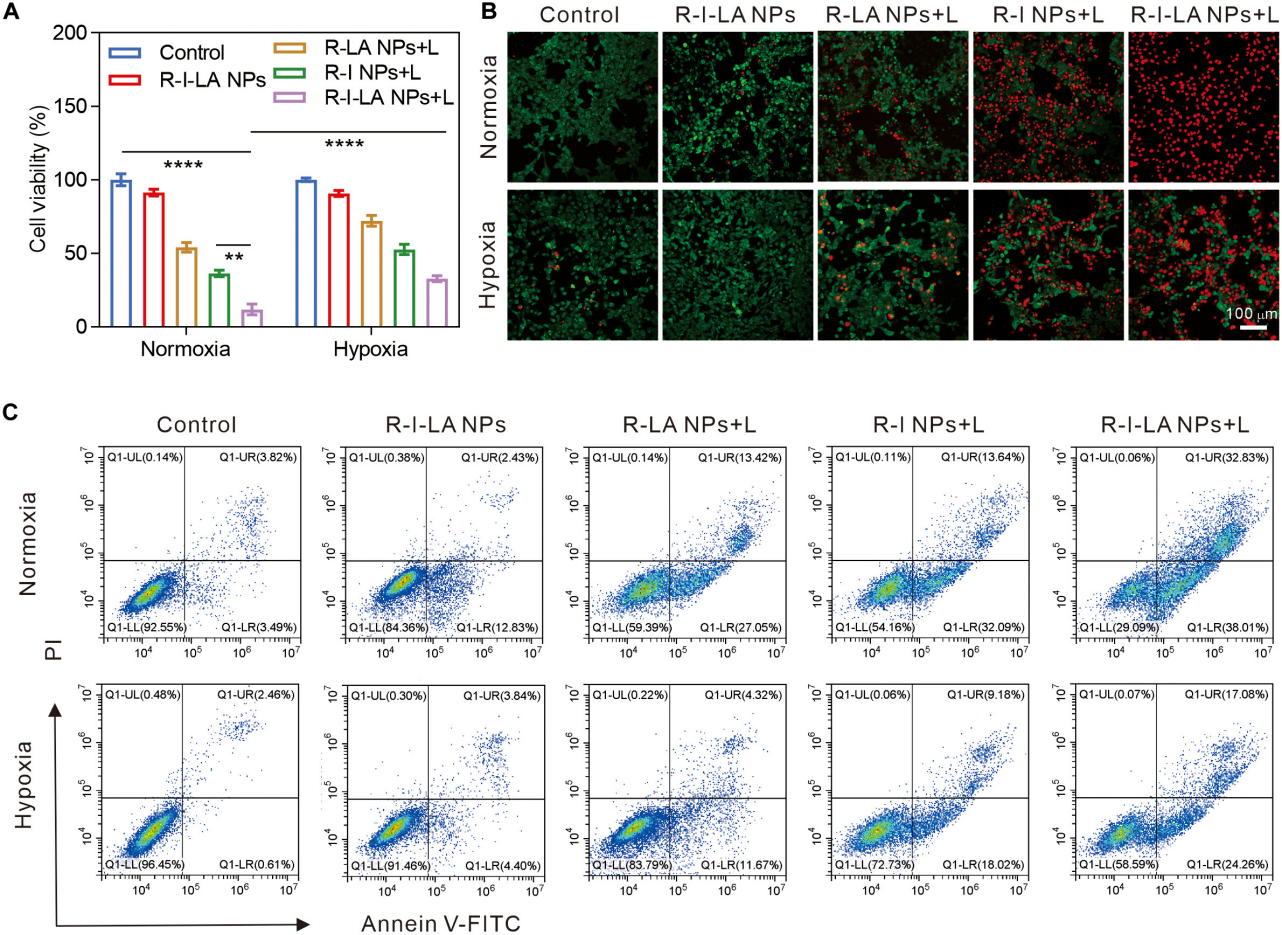
Therapeutic efficacy of R-I-LA NPs in vitro. (A) Cell viability of 4T1 cells after different treatments. (B) CLSM images of cells stained with Calcein-AM/PI staining after different treatments. (C) Apoptosis quantified by FCM analysis. The experiments were repeated thrice independently. ANOVA with Tukey’s post-hoc test. **P < 0.01 and ****P < 0.0001.

Carriers can only achieve clinical translation provided that they are safe enough. Thus, the cytotoxicity of blank PLGA nanoparticles on tumor cells was assessed. The results showed that compared with the control group, the blank PLGA group did not affect the viability of tumor cells, and the cell survival rate was still over 85% after 72 h of incubation. Thus, PLGA carriers have good biosafety (Fig. S8). Effective SDT requires an environment with adequate oxygen; therefore, normoxic and hypoxic groups were established for comparison in this study’s cell experiments. First, we examined the effects of different nanoparticles on the proliferative activity of the 4T1 cells under different oxygen environments using CCK-8 assays (Fig. 4A). The results showed that the control group and R-I-LA NPs group without LIFU irradiation exhibited no significant inhibitory effects on the proliferative activity of 4T1 cells under either normoxic or hypoxic conditions. The cell survival rates of R-LA NPs + L, R-I NPs + L, and R-I-LA NPs + L groups under hypoxic environments were 72.1% ± 3.67%, 52.62% ± 3.48%, and 32.75% ± 2.1%, respectively. However, the R-I-LA NPs + L group exhibited a significant decrease in the survival rate of 4T1 cells (11.84% ± 3.71%) under an oxygen-sufficient environment, which was significantly lower than both the 54.1% ± 3.17% and 36.35% ± 2.21% survival rates exhibited by the R-LA NPs + L and R-I NPs + L groups. These results indicate that irradiation of the nanoparticles with LIFU under an oxygen-sufficient environment exhibited a superior inhibitory effect on tumor cell viability. This effect is because oxygen promotes the production of ROS during SDT, which increases the production of RNS through a series of reactions. Next, we used live-dead staining confocal experiment to deeply examine the cell survival of each group (Fig. 4B). The results demonstrated that nearly all the cells in the R-I-LA NPs + L group exhibited red fluorescence under a normoxic environment, indicating complete cell death. However, the R-I-LA NPs + L group under hypoxic conditions demonstrated an interphase state of red and green fluorescence, which indicated partial death of the cancer cells. The remaining treatment groups exhibited intense red fluorescence under normoxic conditions compared to hypoxic conditions, corroborating the results of CCK-8 cell assays. Finally, we evaluated the difference in the proportion of apoptotic 4T1 cells after SDT treatment in each group using FCM apoptosis assay (Fig. 4C). The results showed that the apoptotic rate of the R-I-LA NPs + L group reached 70.6% ± 2.53% under normoxic conditions, which was significantly higher compared to other groups, indicating that R-I-LA NPs significant facilitated cancer cells’ inactivation after LIFU stimulation, with sufficient oxygen providing better therapeutic effects. This study offers a strong foundation for the subsequent in vivo treatment and provides a basis for the design of mechanisms to increase ECM degradation to improve the hypoxic microenvironment of TNBC.

### Ultrasound-triggered RNS cascade

**Fig. 5.**
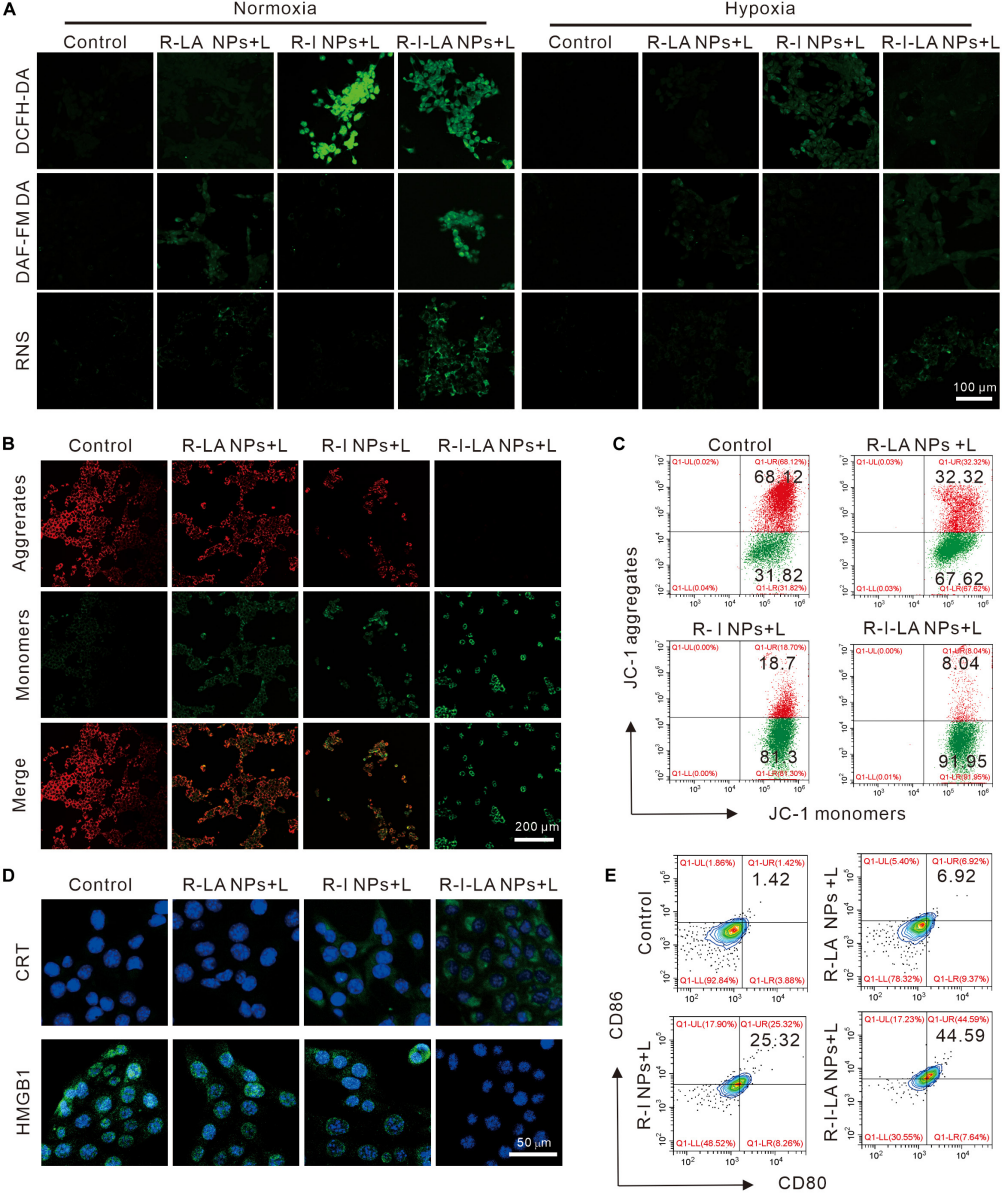
SDT capacity and induction of ICD by R-I-LA NPs in vitro. (A) DCFH-DA probe for evaluating the ROS level; DAF-FM DA probe for the detection of NO generation; RNS probe for the detection of RNS generation. (B) CLSM images and (C) FCM analysis of JC-1-stained 4T1 cells after different treatments. (D) CLSM images of intracellular CRT and HGMB1expression of 4T1 cells after different treatments. (E) FCM analysis of matured DCs (CD11c^+^CD80^+^CD86^+^) after different treatments. The experiments were repeated thrice independently. ANOVA with Tukey’s post-hoc test.

The level of ROS generation is vital given that the fabricated nanoplatforms are designed to improve the SDT efficiency. To measure the level of ROS within cells, we used the DCFH-DA, DAF-FM DA, and RNS dyes that are oxidized by their respective analytes, resulting in green fluorescence emission. Therefore, the intensity of green fluorescence detected in the cells serves as an indicator of the intracellular levels of ROS, NO, and RNS (Fig. 5A).

First, ROS production in each group was detected under CLSM. From Fig. 5A, only a little green fluorescence was detected in the R-I NP + LIFU group, with no detectable fluorescence observed in the other groups under the hypoxic environment. These observations are attributed to the inefficiency of SDT in producing ROS under hypoxic environments, while it exhausts the small amount of ROS that it has in the presence of LA. However, under normoxic conditions, the strongest green fluorescence was observed in the R-I NPs + L group, followed by the R-I-LA NPs + L group. However, almost no green fluorescence was observed in the control and R-LA NPs + L groups. This result demonstrated that ultrasound-triggered ROS generation is oxygen-dependent, and it indicates that LA in R-I-LA NPs consumes part of ROS during NO generation. We then quantified ROS production in each group using FCM (Fig. S9). The results were consistent with the findings observed using CLSM, where the highest proportion of ROS was produced in the R-I NPs + L group under normoxic conditions. Secondly, we observed NO production in each group using CLSM. In Fig. 4A, only the R-I-LA NPs + L group exhibited little green fluorescence under a hypoxic environment, while no detectable fluorescence was observed in other groups. However, under normoxic conditions, the strongest green fluorescence was observed in the R-I-LA NPs + L group, demonstrating that the ROS produced by the ultrasound-triggered SDT effect oxidized LA to generate large amounts of NO. Similarly, NO production in each group was quantified using FCM (Fig. S10), and the results corroborated this of CLSM. Finally, we observed the production of RNS in each group using CLSM. Results illustrated in Fig. 4A indicated that only under normoxic conditions was markedly green fluorescence observed in the R-I-LA NPs + L group, but not in the other groups. This result validates our previous hypothesis that ultrasound triggers the SDT effect of IR780 in R-I-LA NPs to generate ROS, which oxidize NO to generate more toxic RNS, providing the basis for subsequent in vivo SDT combined with RNS treatment.

Studies have reported that RNS can directly oxidize the proenzymatic form of MMPs and convert them into active mature enzymes for ECM degradation. Additionally, RNS can also activate specific signaling pathways, such as the mitogen-activated protein kinase (MAPK) pathway, which regulate the expression and activity of MMPs. Therefore, based on the finding that ultrasound triggers R-I-LA NPs to produce a large amount of RNS, we further examined the expression of MMPs in cells treated with R-I-LA NPs + L using Western blot assays (Fig. S11). The results showed that MMP1 and MMP2 expressed by 4T1 cells were up-regulated after R-I-LA NPs were irradiated with LIFU, suggesting that R-I-LA NPs are vital in inducing the expression and activation of MMPs in vivo.

### Mitochondrial membrane potential detection

Previous mitochondrial targeting experiment results have demonstrated that R-I-LA NPs have excellent mitochondrial targeting performance. Theoretically, R-I-LA NPs penetrating the interior of mitochondria can cause severe mitochondrial damage under LIFU excitation, leading to a decrease or loss of membrane potential. Therefore, we evaluated the mitochondrial membrane potential changes in 4T1 cells treated with R-I-LA NPs + L using the mitochondrial membrane potential detection reagent, JC-1 (Fig. 5B). The results showed that JC-1 exhibited the strongest green fluorescence and weakest red fluorescence in the R-I-LA NPs + L group compared with other groups. This result is attributed to the accumulation of JC-1 within the matrix to produce red fluorescence at high potentials. In contrast, JC-1 forms monomers, thereby emitting green fluorescence at low potentials. Therefore, this result demonstrated that the mitochondria of R-I-LA NPs were severely damaged after LIFU irradiation, resulting in a significant decrease in membrane potential. Subsequently, we quantified the ratio of JC-1 aggregates and monomers using FCM (Fig. 5C). The results for the proportion of JC-1 monomers in the control, R-LA NPs + L, R-I NPs + L, and R-I-LA NPs + L groups were 38.68% ± 7.49%, 73.42% ± 2.52%, 80.43% ± 1.55%, and 92.17% ± 0.24%, respectively (Fig. S12). These results indicate that mitochondria play a significant role in the production of energy for cell survival and contribute substantially in apoptosis. The stimulation of ROS and NO leads to mitochondrial damage, whereas RNS generated through the ROS and NO reactions produce the greatest damage.

### ICD induced by R-I-LA NPs in vitro

SDT combined with RNS synergistically induces ICD in tumor cells, thereby providing an innovative strategy for cancer therapy. The synergistic cytotoxic effect on tumor cells by SDT and RNS induces strong ICD. During ICD, tumor cells release a series of "danger signal" molecules, such as HMGB1 and calreticulin (CRT), which are 2 vital DAMPs, responsible for promoting the maturation of DCs and the occurrence of antitumor immune response [32]. Therefore, the changes of HMGB1 and CRT in 4T1 cells treated with R-I-LA NPs + L were observed using CLSM (Fig. 5D). The CRT results showed that the R-I-LA NPs + L group exhibited significantly enhanced green fluorescence, primarily at the cells’ periphery. This is because CRT is transferred from the ER to the cell surface and exposed during ICD, a process known as evasion of CRT, which is an important marker of ICD development in cells. Additionally, the HMGB1 observation showed that green fluorescence was almost absent in the nucleus of the R-I-LA NPs + L group, while detectable green fluorescence was observed in all other groups. This indicated that irradiation of R-I-LA NPs with LIFU released HMGB1 from the nucleus to the ECM as the ICD process of tumor cells increased in intensity, resulting in the disappearance of green fluorescence in the nucleus. Exonuclear transfer of HMGB1 and the evasion of CRT synergistically promote DC maturation. Subsequently, we examined the effect of different treatments on the maturity of DC cells using FCM (Fig. 5E). The results showed that the DC maturation rate of the R-I-LA NPs + L group was 42.48% ± 1.86%, which was significantly higher than that of the control (1.54% ± 1.02%), R-LA NPs + L (6.02% ± 0.79%), and R-I NPs + L (23.85% ± 2.81%) groups (Fig. S13). These results indicate that R-I-LA NPs activate the antitumor immune response under ultrasound irradiation, thereby corroborating the results that it inhibits TNBC metastasis in vivo.

### Biosafety assay of R-I-LA NPs in vivo

We investigated the survival status of normal mice for 28 days after intravenous R-I-LA NPs tail vein injection. The results indicated no death or weight loss in mice. Additionally, the blood composition and vital organs of the mice were examined at different time points after nanoparticle injection (Fig. S14). The results of routine blood and biochemical tests remained within the reference range, indicating that R-I-LA NPs were not associated with abnormal liver, kidney, and hematopoietic function in vivo. Concurrently, H&E section results showed that there were detectable pathological changes in both the R-I-LA NPs and control groups, indicating that the nanoparticles have a high biosafety in vivo, which is an important prerequisite in both in vivo treatments and future clinical translations (Fig. S15). Next, we studied the effect of PLGA on the TME. The results showed that compared to the control group, the levels of TNF-α and IL-6 at the tumor site fluctuated after the injection of PLGA; however, over time, the levels of TNF-α and IL-6 gradually returned to baseline levels, indicating that the injection of PLGA did not trigger a persistent inflammation (Fig. S16).

### Photoacoustic/fluorescence imaging in vivo and in vitro

Prior to validating the use of R-I-LA NPs for early diagnosis of TNBC in vivo, nanoparticles with varying concentrations were synthesized and their fluorescence and photoacoustic imaging capabilities were examined in vitro.

**Fig. 6.**
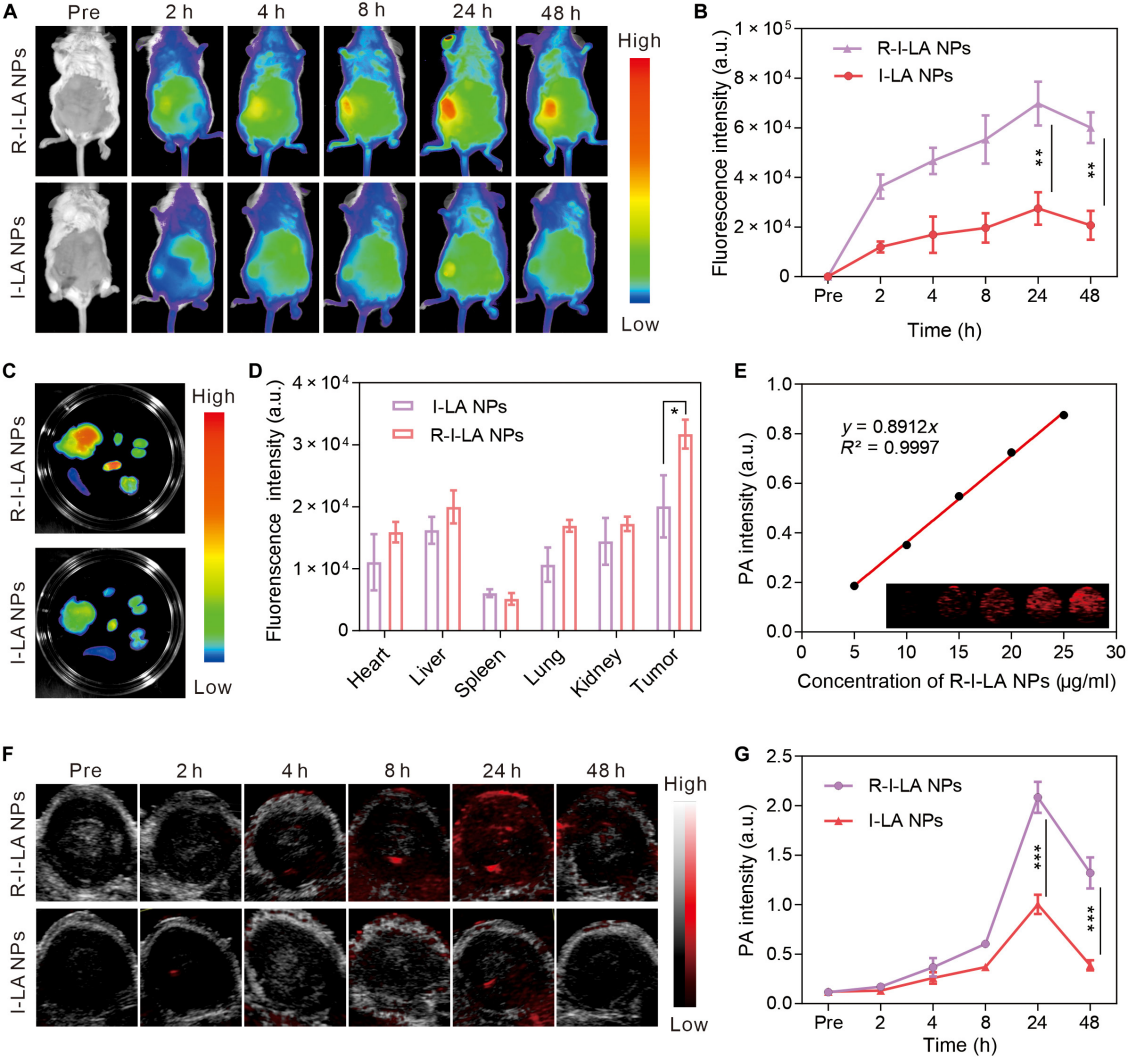
FL/PAI imaging ability of R-I-LA NPs. (A) In vivo FL images of 4T1 tumor-bearing mice at different time points and (B) the corresponding FL intensities of tumors. (C) FL images and (D) corresponding quantitative analysis of isolated organs and tumors. (E) In vitro PAI images and PAI values of R-I-LA NPs at different concentrations. (F) In vivo PAI images of 4T1 tumor-bearing mice at different time points and (G) the corresponding PAI intensities of tumors. The experiments were repeated thrice independently. ANOVA with Tukey’s post-hoc test. *P < 0.05, **P < 0.01, and ***P < 0.001.

First, the in vitro fluorescence imaging performance was evaluated. The results show that R-I-LA NPs maintained excellent fluorescence emission properties even with IR780 encapsulated within their shell. Additionally, the fluorescence signal intensity increased proportionally with the concentration of R-I-LA NPs, indicating a concentration-dependent relationship between nanoparticle levels and fluorescence imaging intensity (Fig. S17). Subsequently, equal concentrations of R-I-LA NPs and I-LA NPs were injected into mice intravenously via the tail vein. The results showed that the fluorescence signal of the R-I-LA NPs group was stronger compared to that of the I-LA NPs group during the entire observation period. This enhanced signal may be attributed to the immune evasion properties conferred by the erythrocyte membrane coating, leading to more efficient aggregation of nanoparticles at the tumor site (Fig. 6A). Additionally, detectable fluorescence signal was observed at the tumor site in the R-I-LA NPs group 2 h postinjection, with the signal reaching its peak at 24 h then regressing (Fig. 6B). Notably, we observed that there was still a strong fluorescence signal at the tumor site in the in vitro tissue, indicating that the R-I-LA NPs group exhibited extended tumor retention effect in the in vivo fluorescence imaging, providing convenience for image-guided therapy and the evaluation of therapeutic effects (Fig. 6C and D). Next, we investigated the photoacoustic imaging performance of R-I-LA NPs. The results showed that the photoacoustic signal intensity of R-I-LA NPs was linearly associated with its concentration (Fig. 6E). Notably, the in vivo PAI results showed that R-I-LA NPs exhibited very strong PAI signal at the tumor site at 24 h postinjection, with the entire in vivo distribution process consistent with the fluorescence imaging results (Fig. 6F and G). Efficient nanoparticles accumulation at the tumor site is a vital prerequisite for the successful implementation of treatment. Consequently, the results of this experiment strengthen our confidence in the efficacy of the in vivo therapeutic approach.

### Antitumor effect of R-I-LA NPs in vivo

**Fig. 7.**
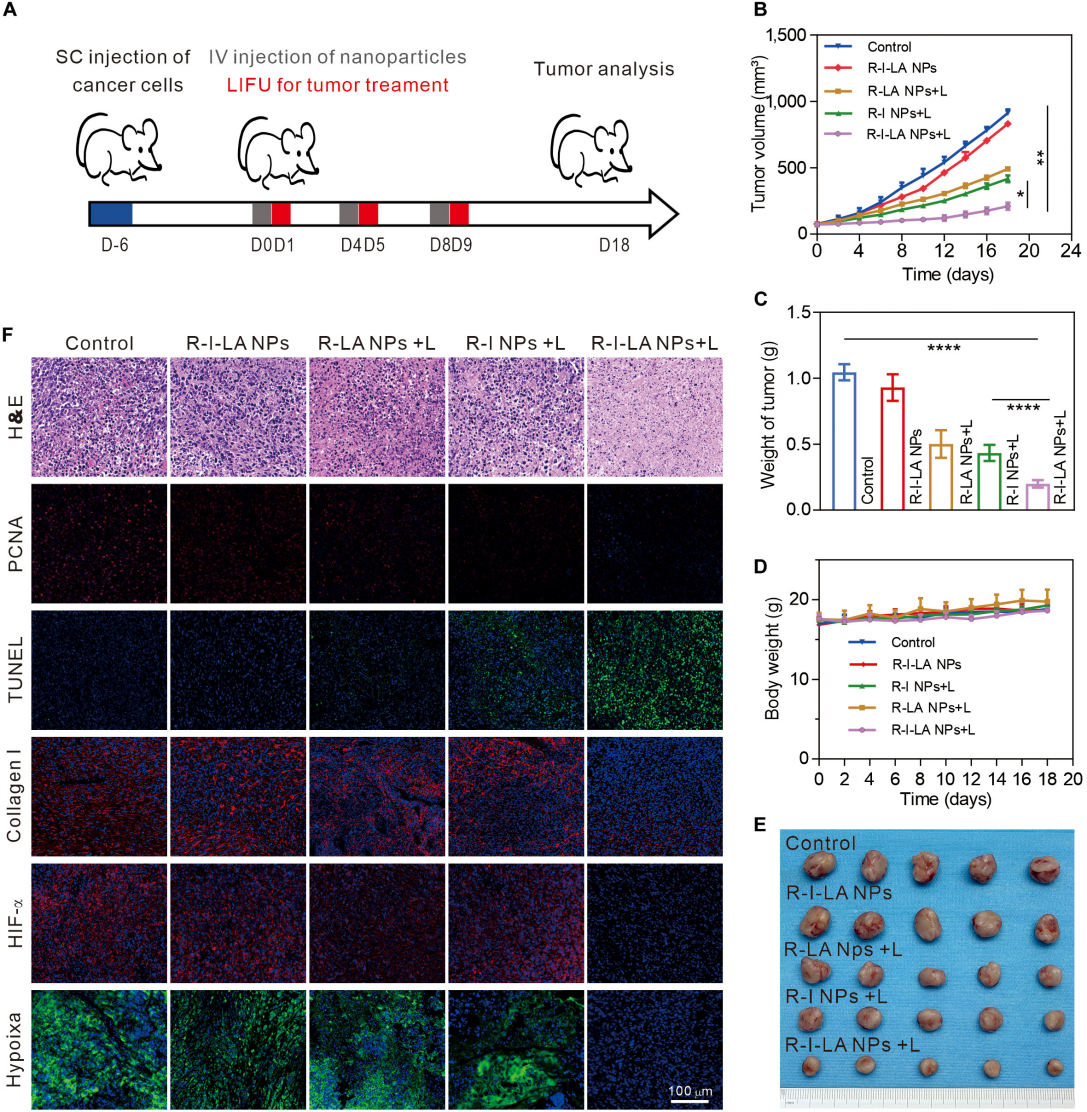
Antitumor efficacy of R-I-LA NPs in vivo. (A) Treatment schedule for the R-I-LA NPs. (B) Tumor growth curves. (C) Tumor weights. (D) Body weight changes. (E) Photographs of tumors dissected from mice. (F) H&E, PCNA, TUNEL, Collagen I, HIF-α, and hypoxia staining of tumors. The experiments were repeated thrice independently. ANOVA with Tukey’s post-hoc test. *P < 0.05, **P < 0.01, and ****P < 0.0001.

The excellent therapeutic efficacy of R-I-LA NPs in vitro and the dual-modality fluorescence/photoacoustic imaging results confirmed the in vivo imaging-guided antitumor potential of R-I-LA NPs. Therefore, we conducted further in vivo antitumor therapy experiments on the nanoparticles. It was observed that the nanoparticles were enriched in the tumor site 24 h after injection; therefore, we selected 24 h as the ideal time point for LIFU irradiation (Fig. 7A). The changes in tumor volume during tumor growth were recorded in all groups (Fig. 7B), which revealed that without LIFU irradiation, the growth rate of tumor volume in the R-I-LA NPs group was higher relative to that in the control group, but only slightly slower compared with that in the control group. However, in the presence of LIFU, the tumor growth rate was significantly inhibited in the R-LA NPs group and the R-I NPs group, and the growth rate of the R-I NPs group was smaller compared with that of the R-LA NPs group, indicating that SDT alone was more effective than NO treatment alone. Most importantly, the R-I-LA NPs + L group had the highest inhibitory effect on tumor growth, which was close to parallel growth, indicating that the effect of 1+1 > 2 would occur when the SDT of IR780 was placed at the same time and space as the NO treatment produced by LA.

At the end of treatment and observation on day 18, the mice were sacrificed and the tumors were dissected and photographed (Fig. 7E). They were then weighed to calculate the relative index of the tumor weight. It was found that the index in the R-I-LA NPs + L group was 0.2 ± 0.03, which allowed objective evaluation of the tumor inhibition effect (Fig. 7C and Fig. S18). During the whole treatment process, the body weight of the mice did not decrease, indicating that the mice survived well without cachexia or serious toxic and side effects (Fig. 7D). Finally, to clarify the inhibition of tumor growth in all groups, we performed pathological staining of the treated tumor tissue (Fig. 7F). H&E images indicated that the morphology and structure of tumor cells in the control group and the R-I-LA NPs group were intact, and no marked nuclear pyknoic necrosis was observed. In contrast, the cells in the other 3 groups exhibited varying degrees of necrosis, among which the cells in the R-I-LA NPs + L group showed the most severe damage. The TUNEL assay is mainly used to detect apoptosis of tumor cells, with a higher proportion of apoptosis corresponding to an intense green fluorescence. The PCNA staining is mainly used to detect the proliferation of tumor cells, with a higher proliferation corresponding to a more intense red fluorescence. The results indicated that the green fluorescence of the R-I-LA NPs + L group was the strongest, while the red fluorescence was weakest in this group, indicating that after LIFU excitation, R-I-LA NPs inhibited the proliferation of many tumor cells and promoted apoptosis.

### Softening of ECM and remission of hypoxia

Type I collagen is the main component of the ECM in solid tumors and is considered to be the main barrier that prevents drug penetration into the tumor [33]. Therefore, immunofluorescence staining was performed on the treated tumor tissue (Fig. 7F). It was observed that only the red fluorescence of labeled type I collagen was markedly reduced in the R-I-LA NPs + L group, indicating marked depletion of type I collagen in the R-I-LA NPs + L group. HIF-1α and hypoxia staining are often conducted to evaluate hypoxic areas in tumor tissues, and our results showed that the hypoxic areas of red HIF-1α and green HIF-1α were undetectable in the R-I-LA NPs + L group. These results indirectly showed that the SDT and RNS cascade generated after R-I-LA NPs excitation by LIFU degraded collagen type I in the ECM to alleviate the tumor hypoxic environment.

**Fig. 8.**
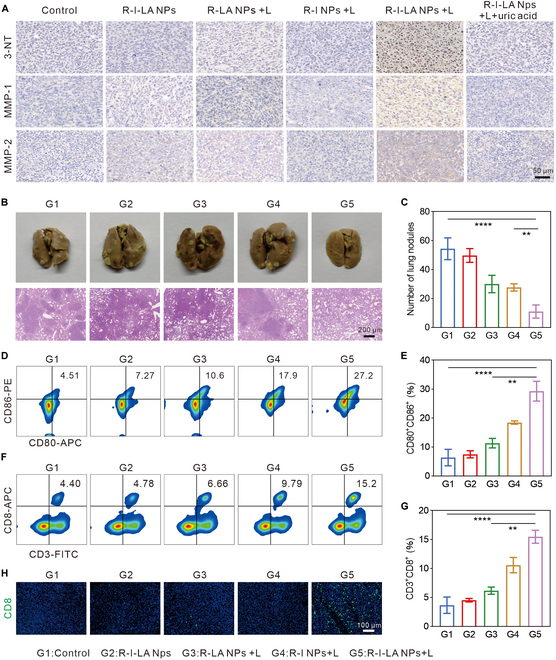
Evaluation of anticancer immune responses caused by R-I-LA NPs. (A) 3-NT, MMP-1, and MMP-2 staining of tumors. (B) Bouin’s trichrome fixed lung tissue and H&E staining images and (C) the number of pulmonary nodules after receiving different treatments. (D) Representative FCM plots of matured DCs in spleens and (E) corresponding quantitative analysis. (F) Representative FCM plots of CD8^+^ T cells in spleens and (G) corresponding quantitative analysis. (H) Immunofluorescence images of CD8^+^ T cells in tumors after different treatments. The experiments were repeated thrice independently. ANOVA with Tukey’s post-hoc test. **P < 0.01 and ****P < 0.0001.

Given the difficult of measuring RNS in tumor tissue, 3-nitrotyrosine (3-NT) is commonly used as a biomarker for in vivo RNS activity evaluation. In this study, immunohistochemical labeling of tumor tissues from different treatment groups, including 3-NT, MMP-1, and MMP-2, was conducted (Fig. 8A). The results showed that the formation of 3-NT was increased and the expression of MMP-1 and MMP-2 was elevated in tumor tissues of the R-I-LA NPs + L group. However, when R-I-LA NPs + L was pretreated with uric acid, the production of 3-NT, MMP-1, and MMP-2 was not markedly different from that of the control group. This is because uric acid can remove RNS generated in vivo, suggesting that the increase of 3-NT, MMP-1, and MMP-2 production was primarily due to the formation of RNS in vivo by R-I-LA NPs stimulated by LIFU. This experiment confirmed that the R-I-LA NPs produced SDT after irradiation with LIFU in vivo. The ROS generated by this process promotes iNOS to oxidize LA to NO, which, in turn, is oxidized to RNS in the environment of ROS, leading to the activation of MMPs to remodel the stiffness of the ECM and relieve hypoxia.

### Activation of antitumor immunity suppressed metastasis in vivo

We previously demonstrated that R-I-LA NPs enhanced the efficacy of SDT by softening ECM to improve tumor hypoxic environment. Additionally, studies have shown that increased ECM stiffness can affect the immune response. The mechanical barrier formed by the overdeposition of collagen in the ECM not only restricts the activation of immune cells within TME but also affects the ability of T cells to home to the tumor site. In this paper, we investigated the specific effects of R-I-LA NPs combined with LIFU treatment on antitumor immunity. On day 35 of treatment, lung tissues were collected from each group and stained with H&E and photographed immediately (Fig. 8B). It was observed that the R-I-LA NPs + L group reduced the number of nodules in the lung metastasis compared with the other groups, but it failed to induce lung metastasis (Fig. 8C). To further determine why R-I-LA NPs inhibited lung metastasis in TNBC, DC maturation and CD8^+^ T cell infiltration in lymph nodes, spleen, and tumor tissues were examined. The results indicated that the DC maturation rate of the R-I-LA NPs + L group was the highest (29.27% ± 3.41%), and the proportion of CD8^+^ T cells in the spleen was increased to 15.47% ± 1.12% (Fig. 8D to G). Immunofluorescence staining of tumor tissue further indicated that CD8^+^ T cell infiltration was highest in the R-I-LA NPs + L group (Fig. 8H). By degrading the tumor mechanical barrier, this self-enhancing design of R-I-LA NPs not only improved hypoxia but also boosted the host-specific antitumor immune, which could significantly inhibit the lung metastasis of TNBC.

## Conclusion

In summary, we have constructed a mechanical immunomodulator (R-I-LA NPs) for remodeling of the ECM, addressing the challenges of poor immune response in TNBC. The R-I-LA NPs, with their biomimetic characteristics, are capable of effectively evading the macrophage phagocytosis and accumulating at the tumor site. R-I-LA NPs enable the spatiotemporal control of the TMM by triggering a LIFU-induced cascade that produces RNS. The process leads to a reduction in ECM stiffness and alleviation of hypoxia, establishing a positive feedback loop that enhances SDT, inducing ICD and stimulating an immune response. ECM remodeling not only markedly enhances antigen presentation efficiency and accelerates DC maturation but also synergistically promotes T cell activation and tumor infiltration, achieving effective cancer cell clearance. Importantly, this effect activates the systemic antitumor immune response and suppresses TNBC metastasis. In conclusion, this study establishes a “sono-gas-mediated mechanical immunity” strategy that can effectively inhibit TNBC proliferation and metastasis.

## Ethical Approval

All animal experiments complied with the relevant ethical standards, and all procedures were approved by the Animal Experimentation Ethics Committee of The Second Affiliated Hospital of Chongqing Medical University.

## Data Availability

The data that support the findings of this study are available on request from the corresponding author. The data are not publicly available due to privacy or ethical restrictions.
