# Supplementary material for: Sono-Gas-Mediated Precise Stiffness Remodeling for Triple-Negative Breast Cancer Mechanical Immunotherapy
Source: Biomater Res. 2025 May 15;29:0207. doi: 10.34133/bmr.0207 (PMC12078941; doi:10.34133/bmr.0207)
Supplement: Supplementary 1 — Figs. S1 to S18 [file bmr.0207.f1.docx]

Supplementary Materials

Sono-gas-mediated Precise Stiffness Remodeling for Triple Negative Breast Cancer Mechanical Immunotherapy

*Yaqin Hu^1, 2, #^, Long Cheng^1, 2, #^, Xun Guo^1, 2^, Min Zheng^1, 2^, Wei Zhang^1, 2^, Xingyue Wang^3^, Rui Tang^1, 2^, Qiaoqi Chen^1, 2^, Yuan Guo^1, 2^, Yang Cao^1, 2^, Zhigang Wang^1, 2^, Haitao Ran^1, 2, *^*

^1^ Department of Ultrasound, the Second Affiliated Hospital of Chongqing Medical University, Chongqing 400010, People's Republic of China

^2^ Chongqing Key Laboratory of Ultrasound Molecular Imaging and Therapy, the Second Affiliated Hospital of Chongqing Medical University, Chongqing 400010, People's Republic of China

^3^ Department of Ultrasound Medicine, Xiangyang Central Hospital of Hubei University, Hubei Province 441021, People's Republic of China

^#^ Y. Hu and L. Cheng contributed equally to this work and shared the first authorship

^*^ Corresponding Author: Haitao Ran, M.D., Department of Ultrasound, the Second Affiliated Hospital of Chongqing Medical University, Chongqing 400010, People's Republic of China. E-mail address: ranhaitao@cqmu.edu.cn; Phone: 86+135-1237-3563; Fax: 86+023+63693348; ORCID: 0000-0002-3820-1970

**Fig. S1.** **The standard curve of free IR780.**

**Fig. S2.** **The amount of IR780 loading and encapsulation, the amount of LA loading and encapsulation.**

**Fig. S3. The standard curve of LA.**

**Fig. S4. SOSG fluorescence intensity at different concentration.**

**Fig. S5. The standard curve of NO.**

**Fig. S6. Changes of NO concentration in R-I-LA NPs under different conditions at different times.**

**Fig. S7. Quantitative analysis of fluorescence intensity in each group.**

**Fig. S8.** **Cell viability of 4T1 cells treated with PLGA nanoparticles.**

**
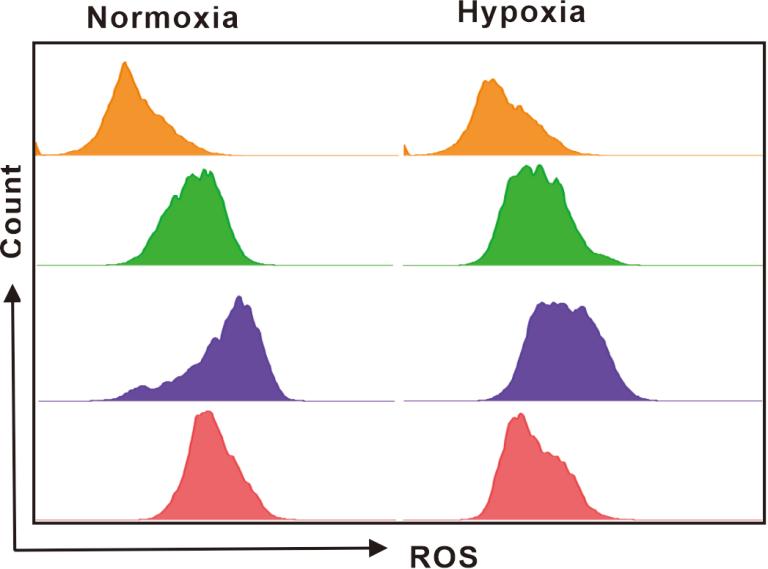
**

**Fig. S9. FCM images of 4T1 cells stained with DCFH-DA (ROS fluorescent probe).**

**
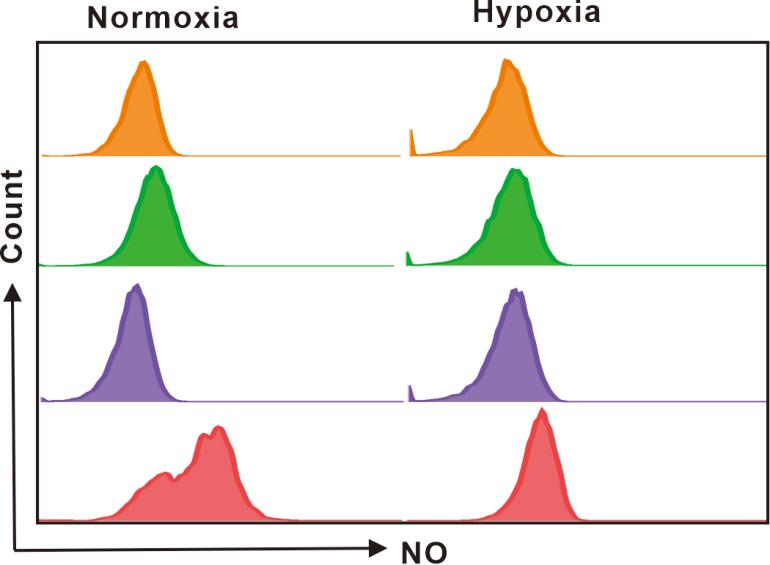
**

**Fig. S10. FCM images of 4T1 cells stained with DAF-FM DA (NO fluorescent probe).**

**
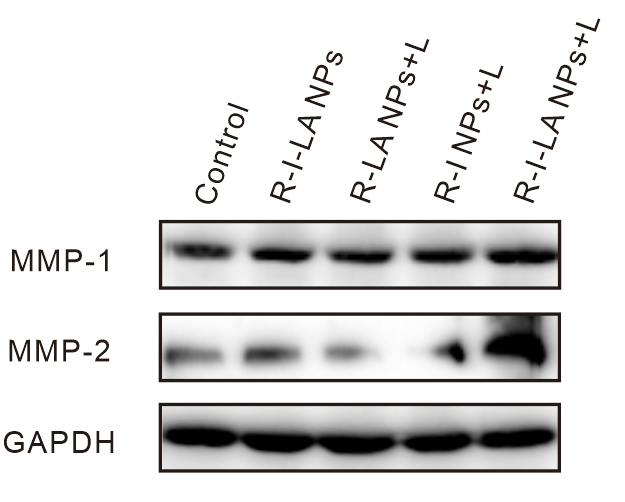
**

**Fig. S11. The increased expressions of MMP-1 and MMP-2 were detected by Western blotting.**

**Fig. S12. Quantitative analysis of JC-1-stained-4T1 cells in different groups.**

**Fig. S13. Quantitative analysis of DC maturation in different groups.**

**Fig. S14. Routine blood and blood biochemistry analysis in mice sacrificed at different time intervals after intravenous administration of R-I-LA NPs.**

**
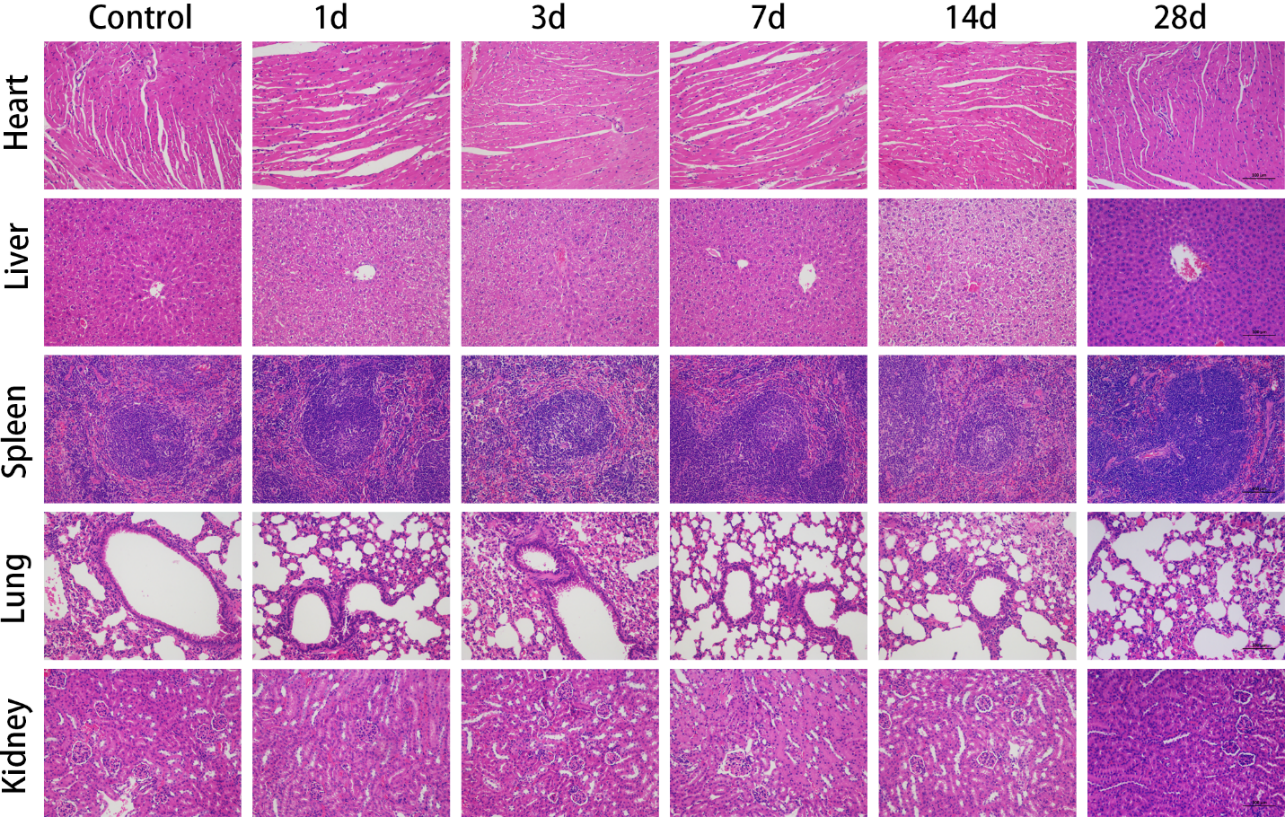
**

**Fig. S15. H&E staining of the major organs in mice sacrificed at different time intervals after intravenous injection of R-I-LA NPs.**

**Fig. S16.** **Cytokine levels in tumors.**

**
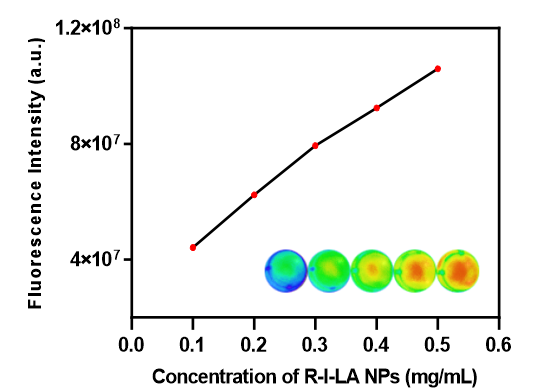
**

**Fig. S17. FL images at elevated concentrations of R-I-LA NPs in vitro.**

**Fig. S18. Tumor weight inhibition rate of tumors.**
